# Light-chain amyloidosis mimicking giant cell arteritis in a bilateral anterior ischemic optic neuropathy case

**DOI:** 10.1186/1471-2415-13-82

**Published:** 2013-12-20

**Authors:** Alberto Neri, Pierangela Rubino, Claudio Macaluso, Stefano A Gandolfi

**Affiliations:** 1 Ophthalmology, University Hospital of Parma, Via Gramsci 14, Parma, PR 43100, Italy

**Keywords:** Ischemic optic neuropathy, Giant cell arteritis, Horton’s disease, Amyloidosis, Light-chain, Monoclonal gammopathy

## Abstract

**Background:**

Herein we report a case of bilateral anterior ischemic optic neuropathy (AION) showing histopathologic evidence of AL-amyloidosis of the temporal arteries. It is known that light-chain (AL) amyloidosis may rarely affect the temporal arteries, mimicking giant cell arteritis, while, to our knowledge, the association between AL-amyloidosis and AION was not previously described.

**Case presentation:**

A 64 year-old woman with AL-amyloidosis secondary to a monoclonal gammopathy of undetermined significance (MGUS) referred to our hospital for acute painless drop of vision due to bilateral AION. Age greater than 50 years, high erythrocyte sedimentation rate (ESR), and bilateral AION were suggestive of giant cell arteritis (GCA). However, a temporal artery biopsy excluded GCA, showing segmental stenosis of the lumen caused by amyloidosis of the artery wall.

**Conclusions:**

The present case shows that AL-amyloidosis may present with AION, high ESR, and temporal artery involvement, mimicking GCA. In patients with monoclonal gammopathies, C-reactive protein may be a more specific index of GCA compared with the ESR. Patient medical history and pathology are crucial for a correct diagnosis.

## Background

Arteritic Anterior Ischemic Optic Neuropathy (Arteritic-AION) is one of the most serious complications of giant cell arteritis (GCA) or Horton’s disease, and it is caused by the occlusion of the posterior ciliary arteries, supplying the optic nerve head (ONH) [1]. Arteritic-AION is the most frequent ophthalmological complication of GCA (81.2%), followed by central retinal artery occlusion (14.1%), retrobulbar ischemic optic neuropathy (7%), and ocular ischemia (1.2%) [1].

Light-chain (AL) amyloidosis, or primary amyloidosis, is a hematological disease characterized by the deposition in tissues of monoclonal light-chain variable-region immunoglobulin fragments, which may involve any tissue or organ. It is usually associated with monoclonal gammopathy of undetermined significance (MGUS) or smoldering myeloma, less frequently with symptomatic multiple myeloma [2]. The vascular system is often involved by the deposition of the amyloidal-proteins, which principally affect the small-caliber vessels [2]. The localization to medium- or large-size vessels is less frequent; nonetheless, deposits of the amyloidal-protein were found in the coronary arteries [3], and arterial thromboembolic events associated with cardiac AL-amyloidosis were reported [4]. AL-amyloidosis of the temporal arteries has also been shown in patients with symptoms and signs suggestive of GCA [5-10].

Here we report a case of bilateral AION showing clinical and laboratory signs suggestive of GCA, which was subsequently excluded by pathology and further laboratory findings. Positivity of the temporal artery biopsy (TAB) for light-chain amyloidosis prompted us to evaluate the possibility that AL-amyloidosis may be a - yet undescribed - cause of bilateral AION, mimicking GCA.

## Case presentation

A 64 year-old woman referred to the emergency service of our department for acute painless drop of vision. The patient reported a complete loss of vision in the right eye and a loss of the inferior visual field in the left eye since waking up in the morning. Past medical history revealed AL-amyloidosis secondary to monoclonal gammopathy of unknown origin (immunoglobulin G light chain k, IgGk) with main involvement of kidneys (nephrosic syndrome), arterial hypertension, and hypercolesterolemia. The diagnosis of AL-amyloidosis had been posed two years before by biopsy of both kidney and periumbilical fat, while negative bone marrow puncture confirmed MGUS. During the previous year the patient had been treated for amyloidosis (one week per month, for 8 months) with dexamethasone (40 mg/day), bortezomib (1.3 mg/m2 two times weekly), and cyclophosphamide (20 mg/Kg once monthly). However, the therapy for amyloidosis was suspended because of hepatic toxicity two months before the presentation of the visual symptoms. At presentation the patient treatment included omeprazole 20 mg/day, ramipril 2.5 mg/day, furosemide 100 mg/day, spironolactone 25 mg/every other day, and atorvastatin 20 mg/day.

**Figure 1:**
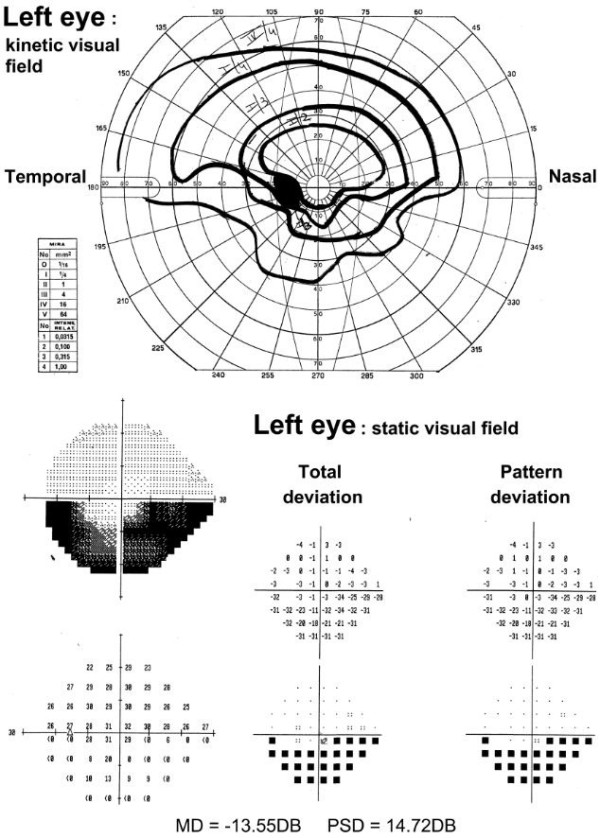
**Visual field examination at presentation.** Kinetic (upside) and static (downside) perimetry of the left eye of the patient at presentation, showing an altitudinal scotoma affecting the inferior visual field. Visual field examination was not executable in the right eye.

Lens corrected visual acuity at presentation was light perception in the right eye and 20/20 in the left eye. Pupil light reflex examination showed a marked relative afferent defect (RAPD) of the right eye. Visual field examination was not executable in the right eye, while in the left eye it revealed an inferior altitudinal scotoma (Figure 1). Biomicroscopy showed normal anterior segment, while at fundoscopy an ONH edema was found in both eyes: in the right eye the edema involved the entire ONH, while in the left eye there was limited involvement of the superior half of the ONH, in keeping with the visual field examination (Figure 1).

Blood analyses were performed at presentation, revealing abnormally high erythrocyte sedimentation rate (ESR = 78 mm/h, laboratory range 2-30 mm), as well as high serum levels of both fibrinogen (745 mg/dL, laboratory range 150-400 mg/dL) and D-dimer (331 ng, laboratory range 0-245 ng). Serum levels of C-reactive protein were within the normal range (CRP = 0.41 mg/dL, laboratory range 0.001-5.0 mg/dL). Recent medical history was negative for claudicatio mandibularis or temporal tenderness.

The patient was admitted to the rheumatology service of our hospital for suspect silent GCA, and high dose corticosteroid therapy was immediately started (1 g methilprednisolone/day i.v. in the first three days, then oral prednisone 50 mg/day). The patient did not report any change of the visual symptoms after starting the corticosteroid therapy. A brain MRI was performed three days after presentation, showing bilateral optic nerve head thickening and multiple chronic ischemic lacunae in sub-cortical and profound white matter. The ultrasound study of the temporal arteries showed tortuosity and segmental stenosis of the arterial lumen, without significant limitation of the blood flow. Ultrasound morphologic alterations typical of GCA (hypo-ecogenic concentric mural thickening) were not found. Carotid Doppler-ultrasound did not identify significant alterations of blood flow.

A biopsy of the right temporal artery was obtained and analyzed histopathologically ten days after presentation, showing diffuse mural infiltration of amyloidal substance, causing the segmental stenosis of the arterial lumen. The amyloid substance showed immunologic positivity for immunoglobulin k light chains and for amyloidal P-substance, and the tunica intima was thickened by fibrosis and calcific deposits, with subocclusion of the vessel. No pathologic signs of GCA were found. Acetylsalicylic acid 100 mg/day p.o. was consequently started, and steroids were slowly tapered.

**Figure 2:**
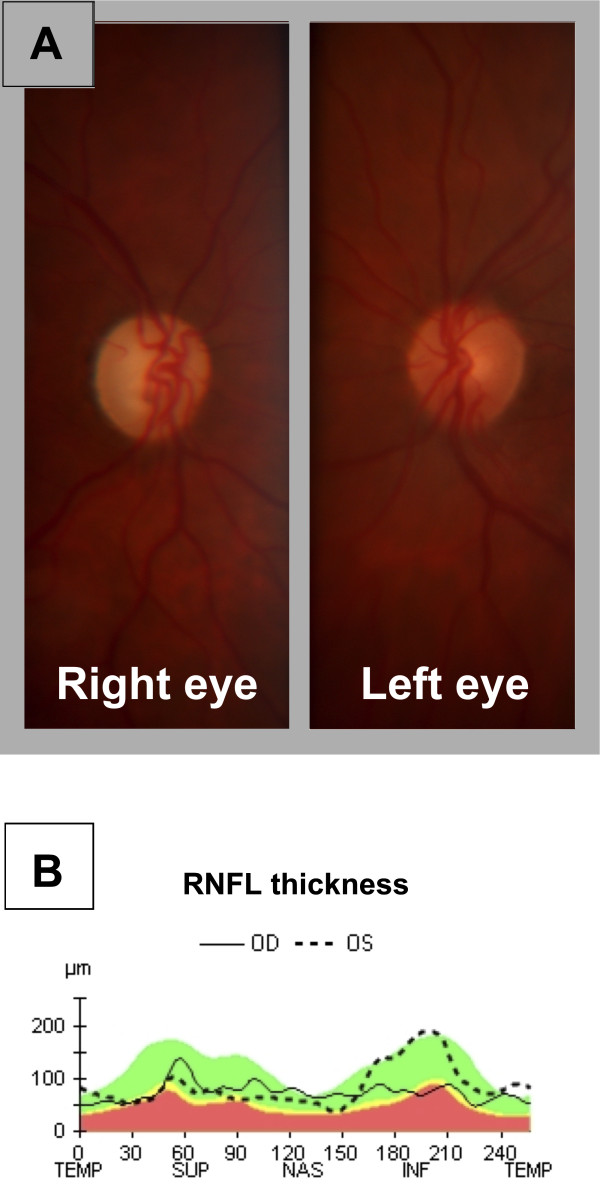
**Optic nerve head evaluation at one**-**year of follow-up. A.** Color pictures of the optic nerve head of the right and left eye of the patient at one-year of follow-up. **B.** Optical coherence tomography evaluation of the Retinal Nerve Fiber Layer thickness (RNFL thickness) at one-year of follow-up, showing relative preservation of the ganglion cell fibers in the inferior quadrants of the left eye, compared with the diffuse reduction of RNFL thickness in the right eye.

An ophthalmological evaluation was repeated one month and one year after presentation. At one-month the ONH edema had almost disappeared, and just a slight masking of the peripapillary vessels was noticeable. At one-year a pallid atrophic ONH was observed in the right eye, and sector atrophy of the ONH was present in the left eye (Figure 2A). Optical coherence tomography (OCT) study of the retinal nerve fiber layer (RNFL) showed a uniformly reduced thickness of the retinal nerve fiber layer (RNFL) in the right eye, and sector reduction of RFNL thickness in the inferior quadrants in the left eye (Figure 2B). Visual acuity and visual field examination remained stable during the follow-up.

## Conclusions

The case herein described presented a problematic differential between arteritic (AAION) and non-arteritic anterior optic neuropathy (NAION). In fact, age greater than fifty years, bilateral eye involvement, and high ESR at presentation were suggestive of a diagnosis of AAION. However, a high ESR is a common laboratory finding in patients with serous monoclonal gammopathies [11], hence its specificity for the diagnosis of AAION may be scarce in these patients. CRP values, instead, were within the normal range in the present case, suggesting that CRP could be a more specific index of AAION in patients with coexisting plasma cell dyscrasias.

The TAB excluded the diagnosis of GCA in the present case, showing diffuse amyloid infiltration of the artery wall. A possible hypothesis explaining the pathogenesis of the optic nerve head ischemia is that the AL-amyloid deposition found in the temporal artery specimen may also have affected the posterior ciliary arteries, causing arterial stenosis and insufficiency, or reduced adaptability to blood pressure variations. As the pathological processes characteristics of GCA commonly involve the posterior ciliary arteries, it is possible that the arteries of this district share with the temporal arteries common histological features causing their preferential susceptibility to GCA. Similarly, the posterior ciliary arteries could share with the temporal arteries a common susceptibility to the deposition of amyloid substance. High blood viscosity due to MGUS, arterial hypertension, and hypercholesterolemia were other factors increasing the risk of NAION in our patient.

The presentation of AION at the awakening in the morning further supports this hypothesis. In fact, blood pressure dips are more commonly encountered during the night, and they are associated to a higher risk of NAION [12-14]. The arterial stenosis combined with a hypotensive dip of the arterial blood pressure could have caused bilateral optic nerve head ischemia in our patient.

AION is rarely bilateral at its exordium [15]. In the present case it can’t be excluded that the patient was not aware of a preexistent monolateral AION until the contralateral eye was involved. However, the appearance of the optic discs at presentation was symmetrical, and indicative for a recent exordium of the ischemia in both the eyes.

Maybe fluorescein angiography (FA) could have provided useful elements for the diagnosis and characterization of the present clinical case. Unfortunately, in our hospital FA is not performed routinely in patients with diagnosis of AION, relying on TAB findings for differential diagnosis between AAION and NAION. When the patient was controlled after the TAB response one month had passed from presentation and the ONH edema had partially regressed, hence it was decided not to perform the angiography.

In conclusion, the present case shows that AL-amyloidosis may present with bilateral AION, mimicking giant cell arteritis not only for the visual symptoms, but also for high ESR values and temporal artery involvement. Moreover, AL-amyloidosis and giant cell arteritis might share similar pathogenetical mechanisms, leading to affect the same small arterial districts. Patient medical history and pathology are crucial for a correct diagnosis.

### Consent

Written informed consent was obtained from the patient for publication of this Case report and any accompanying images. A copy of the written consent is available for review by the Editor of this journal.

## Abbreviations

AAION: Arteritic anterior ischemic optic neuropathy; AION: Anterior ischemic optic neuropathy; AL-amyloidosis: Light-chain amyloidosis; CRP: C-reactive protein; ESR: Erythrocyte sedimentation rate; FA: Fluorescein angiography; GCA: Giant cell arteritis; IgGk: Immunoglobulin G light-chain k; MGUS: Monoclonal gammopathy of undetermined significance; NAION: Non arteritic anterior ischemic optic neuropathy; OCT: Optical coherence tomography; ONH: Optic nerve head; RAPD: Relative afferent pupillary defect; RNFL: Retinal nerve fiber layer; TAB: Temporal arteries biopsy.

## Competing interest

The authors declare that they have no competing interests. Supported in part by funding from University and IMEM-CNR of Parma, Parma, Italy, and from Grant "eRare RHORCOD".

## Authors’ contribution

AN contributed to the clinical management, acquisition and analysis of data, and drafted the manuscript. PR was responsible of the clinical management of the patient. CM have been involved in drafting the manuscript. SAG participated in the design of the case report and revised the manuscript. All authors read and approved the final manuscript.

## Pre-publication history

The pre-publication history for this paper can be accessed here:

http://www.biomedcentral.com/1471-2415/13/82/prepub
